# Systemic Therapy for Unresectable Hepatocellular Carcinoma: Current Landscape and Future Directions

**DOI:** 10.3390/ijms26135994

**Published:** 2025-06-22

**Authors:** Zachary Philippi, Keerthi D. Reddy, Sheza Malik, Zeina Al-Khalil, Nader Dbouk

**Affiliations:** 1 Department of Medicine, Emory University School of Medicine, Atlanta, GA 30322, USA; 2 Rochester General Hospital, Rochester, NY 14621, USA; 3 School of Medicine, American University of Beirut, Beirut 1107 2020, Lebanon; 4 Emory Transplant Center, Atlanta, GA 30322, USA

**Keywords:** hepatocellular carcinoma, systemic therapy, immune checkpoint inhibitors, tyrosine kinase inhibitors, emerging therapies

## Abstract

Hepatocellular carcinoma (HCC), the most common primary liver cancer, remains a leading cause of cancer-related mortality worldwide. Its often-silent progression results in late-stage diagnosis, limiting curative options and necessitating systemic therapy for many patients. The presence of underlying cirrhosis in most cases further complicates treatment decisions. While the approval of sorafenib in 2007 marked a major milestone in systemic therapy for HCC, the treatment landscape has since evolved significantly, particularly with the advent of immune checkpoint inhibitors and anti-angiogenic agents. Combination regimens, such as atezolizumab plus bevacizumab, have demonstrated superior outcomes and are now considered standard first-line options. Despite these advances, efforts to translate insights from HCC’s molecular pathogenesis into personalized treatments have been limited. This narrative review explores the current systemic therapy options for HCC, from first-line to subsequent-line treatments, and highlights emerging strategies, including novel immunotherapies and targeted agents. We emphasize the need for individualized treatment approaches that consider tumor biology, liver function, and performance status, and we outline future directions for research aimed at improving outcomes in this complex and evolving field.

## 1. Introduction

Hepatocellular carcinoma (HCC) is the most common primary malignancy of the liver and remains one of the leading causes of cancer-related mortality worldwide, ranking as the fifth most common cause of cancer death in the United States [1,2]. The insidious nature of HCC often results in late-stage diagnosis, limiting curative treatment options and contributing to poor survival outcomes [3]. Furthermore, most patients with HCC have underlying cirrhosis, which adds complexity to management and influences treatment decisions [4,5,6].

The Barcelona Clinic Liver Cancer (BCLC) staging system is widely used to guide treatment selection based on tumor burden, liver function, and patient performance status [7,8]. While curative options such as surgical resection, local ablation, and liver transplantation are available for patients with early-stage disease, those with intermediate (BCLC-B) or advanced-stage (BCLC-C) disease who are not candidates for transplantation require systemic therapy [7]. Unresectable hepatocellular carcinoma (uHCC) eligible for systemic therapy is defined as disease that is multinodular and unresponsive to locoregional therapies, diffusely infiltrative with bi-lobar involvement, or characterized by portal vein invasion and/or extrahepatic spread, in patients who have preserved liver function and adequate performance status, in alignment with BCLC criteria.

Despite substantial progress in understanding the molecular pathogenesis of HCC, including identification of oncogenic drivers and immune subclasses, few molecular targets have been successfully translated into effective therapies [8]. The approval of sorafenib, a multi-kinase inhibitor, in 2007 marked the first breakthrough in systemic therapy for HCC and led to a paradigm shift in the treatment landscape. In 2020, combination therapy with atezolizumab, an immune checkpoint inhibitor, and bevacizumab, an anti-vascular endothelial growth factor (VEGF) monoclonal antibody, demonstrated superior overall and progression-free survival compared to sorafenib and became the preferred first-line treatment for advanced HCC. In addition, novel immunotherapy-based combinations—including anti-VEGF agents, programmed cell death protein 1 (PD-1) inhibitors, cytotoxic T-lymphocyte-associated protein 4 (CTLA-4) inhibitors, and other immune checkpoint inhibitors—have expanded treatment options. Combinations such as nivolumab plus ipilimumab and pembrolizumab-based regimens have received accelerated approval by the FDA based on promising results from phase II trials [1,8,9,10].

This narrative review provides a comprehensive overview of the current landscape of systemic therapies for hepatocellular carcinoma (HCC), beginning with a focus on the molecular pathogenesis of the disease. We summarize key signaling pathways and genetic alterations that drive hepatocarcinogenesis, including immune-mediated mechanisms. Although advances in understanding HCC biology have identified potential therapeutic targets, translating these insights into effective, personalized treatments remains a challenge.

Building on this foundation, we review current first-, second-, and third-line systemic therapies. We also highlight emerging therapeutic strategies under investigation and discuss the need for personalized approaches that integrate molecular profiling, liver function, and tumor burden to optimize patient outcomes. This review aims to synthesize recent advances and inform clinical practice while identifying future directions for research in HCC treatment.

## 2. Molecular Pathways and Mutations in Hepatocellular Carcinoma Pathogenesis

Hepatocellular carcinoma (HCC) arises from a multifaceted interplay of genetic, epigenetic, metabolic, and microenvironmental alterations. Advances in next-generation sequencing (NGS) have greatly expanded our understanding of the molecular drivers of hepatocarcinogenesis, revealing a heterogeneous landscape marked by recurrent somatic mutations, structural variations, and dysregulated signaling pathways.

A variety of these dysregulated pathways—such as VEGF/VEGFR and TGF-β—are also notably associated with unresectable HCC (uHCC), especially in the presence of macrovascular invasion, extrahepatic spread, or poor differentiation. These features limit surgical candidacy and are frequently enriched in tumors with aggressive molecular phenotypes.

### 2.1. Genomic Alterations

Among the most common genetic alterations in HCC are TERT promoter mutations, present in approximately 60% of tumors. These mutations generate de novo ETS/TCF-binding sites that reactivate telomerase, enabling cellular immortality. Notably, TERT mutations are detectable in about 25% of cirrhotic preneoplastic lesions and frequently result from hepatitis B virus (HBV) integration at the TERT locus [11,12]. In HBV-related HCC, viral integration into oncogenes such as TERT, MLL4, and CCNE1 fosters genomic instability and promotes malignant transformation.

Mutations in TP53 occur in 18–50% of HCCs, particularly the R249S hotspot associated with aflatoxin B1 exposure [13,14]. Loss of TP53 impairs cell cycle regulation, DNA repair, and apoptosis [14]. Activating mutations in CTNNB1 (β-catenin), found in 25–50% of cases, lead to Wnt/β-catenin pathway activation and are typically observed in well-differentiated tumors not linked to viral hepatitis [15]. Mutations in chromatin remodeling genes such as ARID1A and ARID2 (SWI/SNF complex components) are present in 10–20% of tumors, disrupting epigenetic control. ARID2 loss is particularly associated with HCV-related HCC and poor differentiation [16,17]. Additionally, gain-of-function mutations in JAK1 and IL6ST activate the JAK/STAT3 pathway, enhancing inflammation and immune escape [18].

### 2.2. Structural Variations and Viral Integration

Recurrent chromosomal alterations also contribute to HCC pathogenesis. Amplifications in chromosomes 1q and 8q lead to overexpression of oncogenes such as MYC, CCND1, and FGF19, while deletions in 8p and 17p commonly result in the loss of tumor suppressors like TP53 and CDKN2A [19,20]. HBV integration further disrupts host genes, producing chimeric transcripts such as the HBx-LINE1 long non-coding RNA (lncRNA), which activates Wnt signaling and facilitates metastasis [21].

### 2.3. Key Dysregulated Pathways

Several signaling cascades are consistently dysregulated in HCC. The Wnt/β-catenin pathway is activated via CTNNB1 mutations, AXIN1/APC loss, or epigenetic silencing of Wnt antagonists, driving proliferation and chemoresistance [22]. Receptor tyrosine kinase (RTK) pathways, including MET amplification and FGFR4–FGF19 signaling, contribute to invasion and represent actionable targets in select tumors [23]. Activation of the PI3K/Akt/mTOR pathway—through PTEN loss or PIK3CA mutations—supports anabolic growth and metabolic adaptation [24]. The TGF-β pathway plays a dual role, acting as a tumor suppressor early in carcinogenesis but promoting epithelial–mesenchymal transition (EMT) and metastasis in later stages, often through SMAD4 inactivation or TGFBR2 mutation [25].

### 2.4. Immune Evasion and Tumor Microenvironment

Immune escape mechanisms are prominent in HCC. Overexpression of PD-L1 and PD-1 leads to T-cell exhaustion, while regulatory T-cell infiltration dampens anti-tumor immunity [26,27]. These immune checkpoint molecules are also overexpressed in many cases of unresectable HCC. These features form the rationale for immune checkpoint inhibition, with agents such as nivolumab demonstrating clinical efficacy [28].

### 2.5. Epigenetic and Non-Coding RNA Alterations

Epigenetic reprogramming is also central to HCC development. Global DNA hypomethylation coupled with promoter hypermethylation of tumor suppressors like CDKN2A and RASSF1A is common, often mediated by HBx-driven dysregulation of DNA methyltransferases [29,30]. Non-coding RNAs further modulate oncogenic signaling: miR-122 downregulation, seen in up to 70% of HCCs, enhances Wnt activity and metastatic potential, while lncRNAs such as HBx-LINE1, HOTTIP, HULC, and MALAT1 influence tumor progression and hold prognostic value [27,31].

### 2.6. Metabolic Reprogramming

HCC is characterized by a metabolic shift toward aerobic glycolysis (the Warburg effect), driven by HIF-1α and c-MYC, with PKM2 overexpression correlating with poor prognosis. miR-122 regulates glycolytic enzymes and mitochondrial metabolism, further linking metabolic and epigenetic regulation [32,33]. Glutamine addiction, especially in CTNNB1-mutant HCC, sustains nucleotide synthesis through enhanced glutaminolysis [34].

### 2.7. Cancer Stem Cells and the Tumor Microenvironment

Cancer stem cells (CSCs), marked by surface proteins such as EpCAM, CD133, CD24, and CD47, exhibit self-renewal and contribute to therapeutic resistance [35,36,37]. These cells depend on pathways including Wnt/β-catenin and IL-6/STAT3 for stemness maintenance. Notably, NF-κB activation promotes resistance to sorafenib through CD47 upregulation [38]. The surrounding tumor microenvironment—through hypoxia-induced HIF-1α activation and extracellular matrix stiffening—further reinforces stemness via YAP/TAZ signaling and drives resistance mechanisms [39].

Table 1 highlights critical molecular drivers of HCC and their relevance as potential therapeutic targets. Table 2 highlights these pathways that have been linked to uHCC.

### 2.8. Clinical Implications and Therapeutic Challenges

Despite these molecular insights, the translation of HCC genomics into clinical therapies remains limited. Unlike malignancies such as GIST, breast cancer, or melanoma—where driver mutations are routinely targeted—HCC lacks approved therapies for its most frequent mutations, including TERT, CTNNB1, and TP53. The absence of predictive biomarkers, apart from alpha-fetoprotein (AFP) guiding the use of ramucirumab, further constrains precision oncology efforts. Intra-tumoral heterogeneity compounds these challenges, contributing to inconsistent responses and treatment resistance.

Nonetheless, the therapeutic landscape has evolved significantly, with multiple systemic agents approved over the past 15 years based on pivotal phase II and III trials. The following section will provide an overview of current first-, second-, and third-line systemic therapies, key supporting studies, and promising emerging therapies under investigation in early-phase clinical trials.

## 3. Systemic Therapy for HCC

Approximately 50–60% of patients require systemic treatment, either at the time of diagnosis due to advanced disease or following progression after surgery or locoregional therapy [66]. As previously discussed, the Barcelona Clinic Liver Cancer (BCLC) staging system remains the most widely adopted framework for HCC staging, incorporating tumor burden, liver function (Child–Pugh class), performance status, and cancer-related symptoms to stratify patients into five clinical stages and guide treatment decisions [8]. This system has been endorsed by major hepatology and oncology societies in Europe and North America.

Patients are classified from BCLC-0 (very early stage) to BCLC-D (terminal stage). Curative therapies, including surgical resection, liver transplantation, and local ablative techniques (such as radiofrequency or microwave ablation), are preferred for patients with very early (BCLC-0) and early stage (BCLC-A) disease [8]. Intermediate stage (BCLC-B) HCC, characterized by multinodular tumors without vascular invasion or extrahepatic spread, is typically managed with trans-arterial chemoembolization (TACE) [8]. Patients with advanced disease (BCLC-C), defined by the presence of vascular invasion or extrahepatic metastases, are candidates for systemic therapy, whereas those with end-stage disease (BCLC-D) and poor liver function and performance status are best managed with supportive care [8].

Systemic therapy is the mainstay of treatment for patients with advanced HCC who are not eligible for curative or locoregional interventions, offering the only potential for survival benefit in this setting. In some cases, patients with intermediate-stage disease may transition to systemic therapy due to progression or insufficient response to locoregional treatment. For nearly a decade, sorafenib was the sole approved systemic therapy for advanced HCC, with no new agents approved between 2007 and 2016 [67]. However, in recent years, the therapeutic landscape has expanded significantly to include additional tyrosine kinase inhibitors (TKIs), monoclonal antibodies, and immune checkpoint inhibitors (ICIs).

### 3.1. First-Line Therapies

Based on ASCO and BCLC guidelines, combined therapy with atezolizumab and bevacizumab or durvalumab and tremelimumab should be offered as first-line treatment for patients with advanced HCC who have Child–Pugh class A and ECOG performance status 0–1 [8,68]. Both of these regimens demonstrated superior overall survival (OS) when compared to sorafenib, the previous standard of care, in large phase III randomized controlled trials. Atezolizumab is a monoclonal antibody targeting PD-L1 and it enhances immune recognition of tumor cells by preventing PD-L1 from binding to its receptor, restoring T-cell activation and permitting T cells to mount anti-tumor responses [69]. Bevacizumab is an anti-VEGF monoclonal antibody that inhibits angiogenesis by blocking VEGF signaling, thus reducing tumor vascularization and improving immune cell infiltration [70]. The IMbrave150 trial compared atezolizumab plus bevacizumab to sorafenib in patients with unresectable HCC [71]. Atezolizumab plus bevacizumab demonstrated significant OS benefit with a hazard ratio (HR) of 0.66 (95% CI, 0.52–0.85) [45]. The median OS was 19.2 months (95% CI, 17.0–23.7) in the atezolizumab plus bevacizumab group compared to 13.4 months (95% CI, 11.4–16.9) in the sorafenib group [45]. Progression-free survival (PFS) was also significantly improved (6.9 vs. 4.3 months; HR 0.65, 95% CI, 0.53–0.81) [71]. Combination therapy resulted in a 42% lower hazard of death and a 2.5-month increase in median PFS compared to sorafenib. The response rate was 27.3%, with 88% of responders maintaining their response at 6 months [71]. The survival benefit was consistent across various subgroups, including patients with high-risk features such as macrovascular invasion [71]. Additionally, patient-reported outcomes (PROs) from the IMbrave150 trial showed that atezolizumab plus bevacizumab provided clinically meaningful benefits in terms of quality of life, functioning, and disease symptoms compared to sorafenib [72]. These findings established atezolizumab and bevacizumab as the new first-line standard of care for advanced HCC.

Dual checkpoint blockade with tremelimumab (anti-CTLA-4 antibody) and durvalumab (anti-PD-L1 antibody) also demonstrated significant survival benefit and has become an alternative first-line treatment [68]. The HIMALAYA trial was a randomized controlled trial that compared single dose tremelimumab + durvalumab (STRIDE regimen) to sorafenib in patients with advanced HCC [73]. Durvalumab plus tremelimumab significantly improved OS compared to sorafenib alone with HR 0.78 (96.02% CI, 0.65–0.93) [73]. Median OS was 16.43 months (95% CI, 14.16–19.58) with STRIDE, 16.56 months (95% CI, 14.06–19.12) with durvalumab monotherapy, and 13.77 months (95% CI, 12.25–16.13) with sorafenib [73]. Durvalumab monotherapy was noninferior to sorafenib (HR 0.86, 95.67% CI, 0.73–1.03) and PFS was similar across all groups [73]. Both the STRIDE regimen and durvalumab monotherapy were associated with a longer time to deterioration in quality of life, physical functioning, and disease-related symptoms compared to sorafenib [74]. Grade 3/4 treatment-related adverse events (TRAEs) occurred in 25.8% of patients receiving the STRIDE regimen, 12.9% with durvalumab, and 36.9% with sorafenib. The rates of TRAEs leading to discontinuation were 8.2% for STRIDE, 4.1% for durvalumab, and 11.0% for sorafenib [75]. Due to its noninferiority and significantly lower rate of TRAEs compared to sorafenib, durvalumab monotherapy is considered an alternative to sorafenib for patients not appropriate for combined therapy with either atezolizumab plus bevacizumab or durvalumab plus tremelimumab [68,73]. Overall, the HIMALAYA trial established durvalumab plus tremelimumab as an additional first-line treatment option for patients with advanced HCC [8,68].

Clinicians should consider patient-specific factors when choosing between these regimens. Atezolizumab plus bevacizumab requires screening for esophageal varices due to bleeding risks associated with VEGF inhibition [68]. Durvalumab plus tremelimumab may be more appropriate for patients at higher risk for bleeding or thrombosis. Additionally, patients with active or prior autoimmune disease should be evaluated for risk of immune-related adverse effects with atezolizumab and durvalumab plus tremelimumab [68].

In patients who are not candidates for atezolizumab plus bevacizumab or durvalumab plus tremelimumab, tyrosine kinase inhibitors (TKIs), such as sorafenib or lenvatinib, remain the preferred first-line treatment options [8,66,68]. Sorafenib is a multi-kinase inhibitor and acts on VEGF, PDGF, and MAPK. Lenvatinib is also a multi-kinase inhibitor and acts on VEGF, PDGF, FGFR, KIT, and RET. Sorafenib was previously the most widely used therapy for HCC and was recommended as the first-line therapy in all HCC patients based on the landmark SHARP trial, which established the criteria for selecting patients for first-line systemic treatment, taking into account factors such as Child–Pugh liver function stage, tumor burden, and ECOG performance status [76]. This was the first trial to show that systemic therapy with TKIs (specifically sorafenib) could be an effective treatment for HCC; however, the median OS was 10.7 months (HR 0.69, CI 0.55–0.87), which was only 3 months longer than placebo [76]. In 2018, the REFLECT trial was conducted, comparing lenvatinib with sorafenib in a phase 3 non-inferiority trial [77]. The study found that lenvatinib was non-inferior to sorafenib, and median OS was similar at 13.6 months (HR 0.92, CI 0.79–1.06). However, there was an added benefit of a higher objective response rate (ORR) and longer PFS for patients treated with lenvatinib (7.4 months for lenvatinib vs. 3.7–4.3 months with sorafenib) [76,77]. Sorafenib and lenvatinib should be considered first-line therapy in patients that cannot tolerate immunotherapy, including for reasons such as autoimmune disease or liver transplant.

### 3.2. Second-Line Therapies

For patients with advanced HCC who experience disease progression on first-line therapies, second-line therapies should be considered, if appropriate from a functional status and liver function standpoint. Due to the lack of phase III clinical trials, there are minimal data on the optimal sequencing of therapies after progression on atezolizumab plus bevacizumab or durvalumab plus tremelimumab [66]. Most guidelines recommend offering TKIs, such as sorafenib or lenvatinib, first based on the previous treatment hierarchy before combined therapies became first-line [8,66,68]. Cabozantinib or regorafenib may also be considered based on the CELESTIAL and RESORCE studies, respectively, which showed survival benefits in patients who previously tolerated sorafenib and had disease progression [78,79]. Ramucirumab, a monoclonal antibody targeting VEGFR-2, is another FDA-approved second-line therapy for patients with alpha-fetoprotein (AFP) ≥400 ng/mL who are refractory or intolerant to sorafenib, based on the REACH and REACH-2 trials, which demonstrated a significant improvement in OS in this group compared to placebo [80,81].

Based on the ASCO guidelines, additional second-line therapies for advanced HCC include the ICIs, nivolumab or pembrolizumab, alone or in combination with ipilimumab [68]. The CheckMate 040 trial showed patients with advanced HCC had an objective response to nivolumab (monoclonal antibody targeting PD-1) monotherapy; however, the phase III CheckMate-459 trial, which compared nivolumab to sorafenib, did not reach statistical significance for median OS (16.4 vs. 14.7 months, HR 0.85; 95% CI, 0.72–1.02) [82,83]. The nivolumab and ipilimumab combination cohort of the CheckMate-040 trial, which studied nivolumab plus ipilimumab (anti-CTLA4) in patients who progressed on sorafenib, demonstrated an ORR of 32% and median OS of 22.8 months, resulting in accelerated FDA approval as second line-therapy for advanced HCC [84]. Early results from the ongoing phase III CheckMate 9DW trial, which compares nivolumab plus ipilimumab to sorafenib or lenvatinib, have demonstrated a significant OS benefit, with median OS of 23.7 months vs. 20.6 months (HR, 0.79; 95% CI 0.65–0.96), as well as a higher ORR of 36% compared to 13% with lenvatinib or sorafenib (*p* < 0.0001) [85]. These findings suggest that nivolumab plus ipilimumab will likely become a new first-line treatment option for advanced HCC [85,86].

Pembrolizumab, another anti-PD-1 monoclonal antibody, has been studied and approved as a second-line treatment for advanced HCC. Pembrolizumab initially received accelerated FDA approval following the KEYNOTE-224 trial, which demonstrated promising findings in terms of ORR and median OS; however, the follow-up KEYNOTE-240 trial did not meet statistical significance for its primary endpoints of OS and PFS [87,88]. Following these studies, the KEYNOTE-394 trial demonstrated significant OS benefit with pembrolizumab vs. placebo, with median OS of 14.6 compared to 13.0 months (HR, 0.79, 95% CI, 0.63–0.99), along with improved ORR of 13.7% compared to 1.3% [89]. Based on these findings, pembrolizumab remains a second-line treatment option for patients with advanced HCC who have contraindications to or cannot tolerate TKIs and have not previously received an ICI [68].

### 3.3. Third-Line Therapies

Based on ASCO guidelines, third-line therapy may be considered for certain patients with Child–Pugh class A liver function and good performance status, with treatment including a different mechanism of action from prior used therapies [68]. Patients who progress on ICIs or TKIs may benefit from switching to an alternative class of therapy [8,68]. Additionally, cabozantinib, the previously mentioned multi-kinase inhibitor, is recommended as a third-line therapy based on the CELESTIAL trial, which included patients who received sorafenib and up to two prior systemic therapies [78]. This study demonstrated significant OS benefit with cabozantinib of 10.2 months vs. 8.0 months for the placebo (HR 0.76, 95% CI 0.63–0.92) and these results established cabozantinib as an effective second- or third-line treatment option for advanced HCC previously treated with sorafenib [8,68,78]. Given the lack of evidence and phase III clinical trials in this setting, therapy decisions should be individualized through shared decision-making. Furthermore, the clinical significance of third-line therapy remains somewhat unknown as few patients maintain adequate liver function and performance status at this stage, and there are usually limited remaining treatment options [68]. Table 3 summarizes the key trials highlighted in this section. Table 4 compares efficacy outcomes for the various systemic therapies.

### 3.4. Emerging Therapies

Future directions for HCC treatment are focused on several promising areas, including advancements in immunotherapy, targeted therapies, combination treatments, and the use of biomarkers for personalized medicine. As discussed above, many of the therapies were studied in patients with Child–Pugh class A disease and excluded patients with more advanced liver disease. There are some ongoing phase III studies in which patients with Child–Pugh class B or C are included, which will likely impact clinical decision-making of HCC treatment in this subset of patients. The development of resistance to sorafenib and lenvatinib remains an important clinical challenge [90,91]. The identification of patients predisposed to resistance, as well as the establishment of optimal management strategies for TKI-resistant disease, warrant further investigation.

In recent years there has been ongoing discussion regarding the potential role of neoadjuvant and adjuvant systemic therapies in patients undergoing liver resection. Several phase I–III trials are currently underway to address this need [92,93]. Some early results have been promising, with one phase Ib study demonstrating 42% of patients with borderline resectable HCC who received neoadjuvant cabozantinib plus nivolumab had a major pathologic response [94]. In another phase II study, 30% of patients receiving neoadjuvant nivolumab with or without ipilimumab who underwent resection had a major pathologic response [95]. However, at the time of writing this article, the AASLD published a critical update advising against the use of neoadjuvant and adjuvant therapies in patients undergoing liver resection [93]. Further research is needed on the role of neoadjuvant and adjuvant therapies in patients undergoing liver resection.

The development of new molecular-targeted agents continues to be a focus. Agents targeting pathways such as Wnt/β-catenin, c-Myc, and FGFR are being evaluated in clinical trials. Additionally, the combination of targeted therapies with ICIs is being explored to improve treatment efficacy [96,97]. Combining regional therapies like TACE and hepatic arterial infusion chemotherapy (HAIC) with systemic treatments is an area of active research. These combinations aim to improve outcomes in patients with unresectable HCC and increase conversion rates to surgical resection [98]. The identification and utilization of biomarkers such as hypoxia scores and CTNNB1 mutations are being investigated to better personalize treatment and predict patient responses. This approach aims to tailor therapies to individual patient profiles, potentially improving efficacy and reducing adverse effects [98,99]. Epigenetic therapies, including histone deacetylase inhibitors (HDACi), and gene-editing technologies like CRISPR/Cas9, are also emerging as potential treatments for HCC. These therapies target specific genetic and epigenetic alterations in HCC cells [100]. Overall, the future of HCC treatment lies in the integration of these innovative approaches to overcome resistance mechanisms, optimize combination therapies, and enhance personalized treatment strategies. Table 5 below summarizes the ongoing clinical trials for patients with non-resectable HCC compiled from https://clinicaltrials.gov (accessed on 16 April 2025) [101].

## 4. Discussion

The past decade has seen a transformative expansion in systemic therapy options for hepatocellular carcinoma (HCC), enabling more individualized treatment strategies, particularly for patients with intermediate- and advanced-stage disease. Recent guidelines from the American Association for the Study of Liver Diseases (AASLD) strongly recommend systemic therapy for patients with preserved liver function (Child–Turcotte–Pugh class A or B7), an ECOG performance status of 0–1, and Barcelona Clinic Liver Cancer (BCLC) stage C disease. This includes patients with unresectable HCC characterized by diffusely infiltrative bi-lobar involvement, portal vein invasion, and/or extrahepatic spread. Patients with multinodular BCLC B disease who are ineligible for, or have progressed following, locoregional therapies may also benefit from systemic therapy.

While transarterial chemoembolization (TACE) remains the standard first-line therapy for BCLC B disease, increasing evidence supports early transition to systemic therapy in cases of suboptimal response following two TACE sessions, deterioration in liver function, or contraindications to further locoregional treatment. Despite this paradigm shift, there is currently no consensus on the optimal timing or criteria for initiating systemic therapy in these patients, and decision-making remains reliant on clinical judgment.

Immunotherapy-based combinations have reshaped the therapeutic landscape. The combination of atezolizumab and bevacizumab has demonstrated objective response rates of 30–35% and median survival of up to 19 months, supporting its use in selected patients with intermediate-stage disease. First-line therapy is increasingly tailored based on tumor characteristics, liver function, and bleeding risk. Atezolizumab/bevacizumab is preferred in the absence of recent variceal bleeding or high-risk stigmata. All patients should undergo an upper endoscopy prior to therapy initiation; however, the optimal management of patients with large varices remains unclear. For patients who are ineligible for atezolizumab/bevacizumab, the combination of durvalumab and tremelimumab offers a viable alternative. In patients with contraindications to immunotherapy, oral multi-kinase inhibitors such as sorafenib and lenvatinib remain acceptable options. For select CTP B patients, single-agent immunotherapy or tyrosine kinase inhibitors may be cautiously considered, although data in this subgroup remain limited.

Subsequent-line therapies are selected based on prior treatments, liver function, and clinical trial eligibility. Patients who progress on first-line immunotherapy may receive sorafenib or lenvatinib, while additional approved options include cabozantinib, regorafenib, ipilimumab plus nivolumab, and ramucirumab for patients with AFP ≥ 400 ng/mL. Treatment choice should be informed by prior toxicities, performance status, and drug availability. Notably, immune checkpoint inhibitors are contraindicated in patients with recurrent HCC following liver transplantation, due to the high risk of graft rejection and mortality; in these patients, sorafenib or lenvatinib remain the agents of choice.

Despite these advances, important gaps remain. The absence of predictive biomarkers limits the ability to personalize therapy, and patients with Child–Pugh class B cirrhosis continue to be underrepresented in clinical trials, hindering the generalizability of evidence-based recommendations. Future research must prioritize the inclusion of real-world populations, including those with hepatic dysfunction, macrovascular invasion, and extrahepatic spread, to inform clinical decision-making. Recent studies have also highlighted promising systemic regimens capable of downstaging unresectable hepatocellular carcinoma to resectable disease, highlighting the potential for these systemic therapies to potentially achieve cure. These treatment regimens warrant further investigation in larger, randomized controlled trials with extended follow-up to assess long-term outcomes [102,103,104].

In parallel, the integration of early advance care planning (ACP) into HCC management is essential. A new diagnosis of HCC often represents a turning point in a patient’s illness trajectory, necessitating discussions around prognosis, treatment goals, and quality of life. While ACP may be less relevant for patients with early-stage disease, it should be standard practice for those with advanced disease or receiving palliative-intent therapy. Early and iterative ACP can help align care with patient values, reduce unnecessary interventions, and alleviate emotional and financial burdens for patients and their families.

In conclusion, the systemic treatment landscape for unresectable HCC continues to evolve rapidly, improving survival and response rates in patients with intermediate and advanced disease. Timely initiation of systemic therapy, strategic sequencing, and the integration of patient-centered care—including ACP—are key components of optimal HCC management. Future research must aim to refine transition points from locoregional to systemic therapy, develop predictive biomarkers, and expand therapeutic options for underrepresented patient populations.

## Figures and Tables

**Table 1:** Key genetic alterations and dysregulated pathways in hepatocellular carcinoma with potential therapeutic relevance.

Pathway	Gene/Mutation	Function	Targetable
Telomerase Activation	TERT promoter mutations/HBV integration	Reactivates telomerase, bypasses senescence	Imetelstat (telomerase inhibitor) in trials; no approved therapy yet [40]
DNA Damage Response	P53 (R249S hotspot), BRCA1/2	Impairs DNA repair, apoptosis; BRCA loss causes genomic instability	PARP inhibitors (olaparib) in trials for BRCA+ HCC; no direct TP53 therapies [41]
Wnt/β-catenin	CTNNB1, AXIN1, APC	Wnt activation, proliferation, chemoresistance	Porcupine inhibitors (LGK974) and β-catenin disruptors in preclinical [42]
Chromatin Remodeling	ARID1A, ARID2, SMARCA4	Impaired epigenetic control, chromatin instability	HDAC inhibitors (panobinostat) and EZH2 inhibitors in trials [43]
JAK/STAT	JAK1, IL6ST, STAT3 amplification	STAT3 activation, inflammation, immune evasion	STAT3 antisense oligonucleotides (AZD9150) and JAK inhibitors (ruxolitinib) [44,45]
RTK (MET, FGFR, EGFR)	MET amp, FGF19 amp, EGFR mutations	Proliferation, angiogenesis, metastasis	MET inhibitors (tepotinib), [46] FGFR inhibitors (pemigatinib), EGFR (erlotinib) [47,48]
PI3K/Akt/mTOR	PTEN loss, PIK3CA, AKT1 mutations	Growth, metabolic reprogramming	mTOR inhibitors (everolimus), [49] AKT inhibitors (ipatasertib) in trials [50]
TGF-β	SMAD4 inactivation, TGFBR2 mutations	EMT, metastasis, immunosuppression in late stages	Galunisertib (TGF-βR1 inhibitor) [51] & fresolimumab (antibody) in trials [52]
Immune Checkpoints	PD-L1/PD-1, CTLA-4, LAG3, TIM3	T-cell exhaustion, immune evasion	Approved: nivolumab (PD-1), ipilimumab (CTLA-4); LAG3 inhibitors in trials [53]
Epigenetic Regulation	CDKN2A, RASSF1A hypermethylation	Silences tumor suppressors, promotes cell cycle progression	Hypomethylating agents (azacitidine) and HDAC inhibitors in trials [54]
Metabolic Reprogramming	HIF-1α, c-MYC, IDH1/2, PKM2	Aerobic glycolysis, glutamine dependency, lipid synthesis	IDH1 inhibitors (ivosidenib), PKM2 modulators in trials [55]
Cancer Stem Cell	EpCAM, CD133, CD47, NANOG, ALDH1A1	Self-renewal, therapy resistance, immune evasion	Anti-CD47 (magrolimab) and ALDH inhibitors in early trials [56]
Tumor Microenvironment	YAP/TAZ, CAFs, HIF-1α, CXCL12	CSC niche, angiogenesis, immune suppression	YAP/TAZ inhibitors (verteporfin) [57], CXCR4 antagonists (plerixafor) in preclinical [58]
Hedgehog Signaling	SMO, GLI1/2 mutations	Stemness, desmoplasia, tumor-stroma crosstalk	Glasdegib (SMO inhibitor) in phase I/II trials [59]
Notch Signaling	NOTCH1/2/3, JAG1 overexpression	Differentiation blockade, angiogenesis	Gamma-secretase inhibitors (AL101) in preclinical [60]
RAS/RAF/MEK/ERK	KRAS/NRAS, BRAF V600E	Proliferation, survival, metastasis	MEK inhibitors (trametinib) in trials for BRAF-mutant HCC [61]
VEGF/VEGFR	VEGFA amp, VEGFR2 mutations	Angiogenesis, immune suppression	Approved: sorafenib, lenvatinib, ramucirumab (VEGFR2) [62]
Ferroptosis	GPX4 downregulation, SLC7A11 loss	Lipid peroxidation-driven cell death	Ferroptosis inducers (erastin, sulfasalazine) in preclinical [63]
Autophagy	ATG5/7, BECN1 mutations	Stress adaptation, chemoresistance	Chloroquine/hydroxychloroquine in trials with chemo [64]
Ubiquitin-Proteasome	FBXW7 mutations	Stabilizes oncoproteins (c-MYC, mTOR)	Proteasome inhibitors (bortezomib) in early trials [65]

**Table 2:** Key genetic alterations and their link to unresectable HCC.

Genetic Alteration/Pathway	Association with Unresectable HCC
TP53 Mutation (p53 pathway)	TP53-mutated tumors show high rates of vascular invasion and poor prognosis, often being advanced.
RAS/RAF/MAPK Pathway	Activation of RAS/MAPK (e.g., *KRAS* mutation or FGF19–FGFR4 amplification) is linked to extrahepatic metastasis and aggressive disease.
PI3K/Akt/mTOR Pathway	Frequently active in HCC (~50%); drives proliferation, motility, and angiogenesis, thereby facilitating tumor progression.
TGF-β Signaling	Elevated TGF-β activity promotes EMT, invasion, and metastasis; associated with vascular invasion in HCC.
VEGF/VEGFR (Angiogenesis)	High VEGF levels correlate with microvascular invasion and metastasis in advanced HCC.
Immune Checkpoints (PD-1/PD-L1, CTLA-4)	PD-L1 overexpression is tied to vascular invasion and advanced stage in HCC.
Tumor Microenvironment (CAFs, TAMs)	Pro-tumor stroma secretes factors (TGF-β, etc.) that enhance HCC invasion and spread.
Hedgehog Signaling	Hh pathway activation is associated with worse prognosis and more advanced, invasive HCC.
Hippo Pathway (YAP activation)	YAP overexpression correlates with poor survival, intrahepatic metastases, and vascular invasion.
Epigenetic Alterations (e.g., ARID1A)	Mutations like *ARID1A* are linked to larger tumors with microvascular invasion.

**Table 3:** Summary of key clinical trials in systemic therapy for advanced HCC.

Trial Name	Type of Trial	Primary Outcomes	Secondary Outcomes	Patient Population	Number of Patients	Key Findings
IMbrave150 [70]	Randomized Controlled Trial, Open-label	Overall Survival (OS), Progression-Free Survival (PFS)	Objective Response Rate (ORR), Safety, Patient-Reported Outcomes (PROs)	Unresectable HCC, no prior systemic therapy, Child–Pugh class A, ECOG PS 0-1	501 (336 atezolizumab + bevacizumab, 165 sorafenib)	Atezolizumab + bevacizumab significantly improved OS (HR 0.66, *p* < 0.001) and PFS (HR 0.65, *p* < 0.001) compared to sorafenib. ORR was 29.8% for atezolizumab + bevacizumab vs. 11.3% for sorafenib.
HIMALAYA [73]	Randomized Controlled Trial, Open-label	Overall Survival (OS)	Objective Response Rate (ORR), Safety, Patient-Reported Outcomes (PROs)	Unresectable HCC, no prior systemic therapy, Child–Pugh class A, ECOG PS 0-1	1171 (393 STRIDE, 389 durvalumab, 389 sorafenib)	STRIDE regimen (tremelimumab + durvalumab) significantly improved OS (HR 0.78, *p* = 0.0035) compared to sorafenib. ORR was 20.1% for STRIDE vs. 5.1% for sorafenib. Durvalumab monotherapy was noninferior to sorafenib for OS (HR 0.86, 95.67% CI, 0.73–1.03).
SHARP [76]	Randomized Controlled Trial, Double-blind	Overall Survival (OS)	Time to Progression (TTP), Disease Control Rate (DCR), Safety	Advanced HCC, Child–Pugh class A, ECOG PS 0-2	602 (299 sorafenib, 303 placebo)	Sorafenib significantly improved OS (HR 0.69, *p* < 0.001) compared to placebo. Median OS was 10.7 months for sorafenib vs. 7.9 months for placebo.
REFLECT [77]	Randomized Controlled Trial, Open-label	Overall Survival (OS)	Progression-Free Survival (PFS), Objective Response Rate (ORR), Safety	Unresectable HCC, no prior systemic therapy, Child–Pugh class A, ECOG PS 0-1	954 (478 lenvatinib, 476 sorafenib)	Lenvatinib was non-inferior to sorafenib for OS (HR 0.92, 95% CI, 0.79–1.06). Median OS was 13.6 months for lenvatinib vs. 12.3 months for sorafenib.
CELESTIAL [78]	Randomized Controlled Trial, Double-blind	Overall Survival (OS)	Progression-Free Survival (PFS), Objective Response Rate (ORR), Safety	Advanced HCC, previously treated with sorafenib, Child–Pugh class A, ECOG PS 0-1	707 (470 cabozantinib, 237 placebo)	Cabozantinib significantly improved OS (HR 0.76, *p* = 0.005) compared to placebo. Median OS was 10.2 months for cabozantinib vs. 8.0 months for placebo.
RESORCE [79]	Randomized Controlled Trial, Double-blind	Overall Survival (OS)	Progression-Free Survival (PFS), Objective Response Rate (ORR), Safety	Advanced HCC, previously treated with sorafenib, Child–Pugh class A, ECOG PS 0-1	573 (379 regorafenib, 194 placebo)	Regorafenib significantly improved OS (HR 0.63, *p* < 0.001) compared to placebo. Median OS was 10.6 months for regorafenib vs. 7.8 months for placebo.
REACH [80]	Randomized Controlled Trial, Double-blind	Overall Survival (OS)	Progression-Free Survival (PFS), Objective Response Rate (ORR), Safety	Advanced HCC, previously treated with sorafenib, Child–Pugh class A, ECOG PS 0-1	565 (283 ramucirumab, 282 placebo)	Ramucirumab did not significantly improve OS compared to placebo (HR 0.87, *p* = 0.14).
REACH-2 [81]	Randomized Controlled Trial, Double-blind	Overall Survival (OS)	Progression-Free Survival (PFS), Objective Response Rate (ORR), Safety	Advanced HCC, previously treated with sorafenib, AFP ≥ 400 ng/mL, Child–Pugh class A, ECOG PS 0-1	292 (197 ramucirumab, 95 placebo)	Ramucirumab significantly improved OS (HR 0.71, *p* = 0.0199) compared to placebo. Median OS was 8.5 months for ramucirumab vs. 7.3 months for placebo.
CheckMate-040 [82]	Phase I/II, Open-label	Safety, Objective Response Rate (ORR)	Overall Survival (OS), Progression-Free Survival (PFS)	Advanced HCC, previously treated with sorafenib, Child–Pugh class A, ECOG PS 0-1	262 (48 nivolumab, 214 nivolumab + ipilimumab)	Nivolumab showed an ORR of 14.3% and a median OS of 15.1 months. Nivolumab + ipilimumab showed an ORR of 32% and a median OS of 22.8 months.
CheckMate-459 [83]	Randomized Controlled Trial, Open-label	Overall Survival (OS)	Progression-Free Survival (PFS), Objective Response Rate (ORR), Safety	Advanced HCC, no prior systemic therapy, Child–Pugh class A, ECOG PS 0-1	743 (371 nivolumab, 372 sorafenib)	Nivolumab did not significantly improve OS compared to sorafenib (HR 0.85, *p* = 0.0752). Median OS was 16.4 months for nivolumab vs. 14.7 months for sorafenib.
CheckMate-9DW [85]	Randomized Controlled Trial, Open-label	Overall Survival (OS)	Progression-Free Survival (PFS), Objective Response Rate (ORR), Safety	Advanced HCC, no prior systemic therapy, Child–Pugh class A, ECOG PS 0-1	668 (335 nivolumab + ipilimumab, 333 lenvatinib or sorafenib)	Nivolumab + ipilimumab significantly improved OS (HR 0.79, *p* = 0.0180) compared to sorafenib. Median OS was 23.7 months for nivolumab + ipilimumab vs. 20.6 months for lenvatinib or sorafenib.
KEYNOTE-224 [87]	Phase II, Open-label	Objective Response Rate (ORR)	Overall Survival (OS), Progression-Free Survival (PFS), Safety	Advanced HCC, previously treated with sorafenib, Child–Pugh class A, ECOG PS 0-1	104 (104 pembrolizumab)	Pembrolizumab showed an ORR of 17% and a median OS of 13.9 months.
KEYNOTE-240 [88]	Randomized Controlled Trial, Double-blind	Overall Survival (OS), Progression-Free Survival (PFS)	Objective Response Rate (ORR), Safety	Advanced HCC, previously treated with sorafenib, Child–Pugh class A, ECOG PS 0-1	413 (278 pembrolizumab, 135 placebo)	Pembrolizumab did not significantly improve OS (HR 0.78, *p* = 0.0209) or PFS (HR 0.72, *p* = 0.0002) compared to placebo (statistically significant but did not meet the primary endpoint)
KEYNOTE-394 [89]	Randomized Controlled Trial, Double-blind	Overall Survival (OS)	Progression-Free Survival (PFS), Objective Response Rate (ORR), Duration of Response (DOR)	Advanced HCC, previously treated with sorafenib, Child–Pugh class A, ECOG PS 0-1	453 (300 pembrolizumab, 153 placebo)	Pembrolizumab significantly improved median OS compared to placebo (14.6 vs. 13.0 months (HR 0.79, *p* = 0.0180) as well as PFS and ORR.

**Table 4:** Efficacy outcomes of systemic therapy for HCC.

Trial	Therapy	Overall Survival (Months)	Progression-Free Survival (Months)	Objective Response Rate (%)
IMbrave150 [70]	Atezolizumab + Bevacizumab	19.2	6.8	27.3
HIMALAYA [73]	Tremelimumab + Durvalumab	16.4	3.8	20.1
SHARP [76]	Sorafenib	10.7	5.5	2.0
REFLECT [77]	Lenvatinib	13.6	7.4	24.1
CELESTIAL [78]	Cabozantinib	10.2	5.2	4.0
RESORCE [79]	Regorafenib	10.6	3.1	11.0
REACH [80]	Ramucirumab	9.2	2.8	4.6
REACH-2 [81]	Ramucirumab	8.5	2.8	4.6
CheckMate-040 [82]	Nivolumab	15.0	4.0	15.0–20.0
CheckMate-459 [83]	Nivolumab	16.4	3.7	15.0
CheckMate-9DW [85]	Nivolumab + Ipilimumab	23.7	9.1	36.0
KEYNOTE-224 [87]	Pembrolizumab	12.9	4.9	17.0
KEYNOTE-240 [88]	Pembrolizumab	13.9	3.0	18.3
KEYNOTE-394 [89]	Pembrolizumab	14.6	2.6	12.7

**Table 5:** Summary of ongoing NIH clinical trials for non-resectable HCC.

NCT Number	Study Title	Study Status	Interventions	Primary Outcome Measures	Enrollment	Study Type	Study Design	Start Date	Completion Date
NCT06261138	Survival Analysis: TACE vs. Combination Therapy in HCC	Completed	DRUG: Transarterial chemoembolization|OTHER: Systemic treatment	Progression-free survival (PFS) per mRECIST: The duration from treatment initiation to PD in patients who cannot undergo surgery, or to the date of postoperative relapse in patients who receive surgery, or death for any reason, whichever occurs first (according to mRECIST), 12–48 months	279	Observational	Observational Model: Time Perspective: p	1 February 2019	31 October 2023
NCT05630937	Study on Safety and Efficacy of NMS-01940153E in Adult Patients With Unresectable Hepatocellular Carcinoma (HCC) Previously Treated With Systemic Therapy	Completed	DRUG: NMS-01940153E	Drug-related dose limiting toxicities (DLTs) (phase I), Cycle 1 (28 days); objective response rate (ORR) (phase II), within two years	31	Interventional	Allocation: NA|Intervention Model: SEQUENTIAL|Masking: NONE|Primary Purpose: TREATMENT	13 November 2020	6 August 2024
NCT00812175	Global Investigation of Therapeutic Decisions in Hepatocellular Carcinoma and of Its Treatment With Sorafenib	Completed	DRUG: Sorafenib (Nexavar, BAY43-9006)	The safety of Nexavar in all patients with unresectable HCC who are candidates for systemic therapy and in whom a decision to treat with Nexavar has been made under real-life practice conditions, at each follow-up visit, every 2–4 months on average	3371	Observational	Observational Model: Time Perspective: p	January 2009	April 2012
NCT06680258	A Study of TPST-1120 With Atezolizumab Plus Bevacizumab in Patients With Unresectable or Metastatic HCC Not Previously Treated With Systemic Therapy	NOT_YET_RECRUITING	DRUG: TPST-1120|BIOLOGICAL: Atezolizumab|BIOLOGICAL: Bevacizumab	Overall survival (OS), defined as the time from randomization to death from any cause up to 60 months; the time from randomization to death from any cause up to 60 months	740	Interventional	Allocation: RANDOMIZED|Intervention Model: PARALLEL|Masking: QUADRUPLE (PARTICIPANT, CARE_PROVIDER, INVESTIGATOR, OUTCOMES_ASSESSOR)|Primary Purpose: TREATMENT	29 March 2025	December 2029
NCT00901901	Nexavar-Tarceva Combination Therapy for First Line Treatment of Patients Diagnosed With Hepatocellular Carcinoma	Completed	DRUG: Sorafenib (Nexavar, BAY43-9006)|DRUG: Erlotinib (Tarceva)|DRUG: Placebo	Overall survival (OS): Overall survival (OS) was defined as the time from date of randomization to death due to any cause; from randomization of the first patient until 34 months or date of death of any cause whichever came first	732	Interventional	Allocation: RANDOMIZED|Intervention Model: PARALLEL|Masking: DOUBLE (PARTICIPANT, INVESTIGATOR)|Primary Purpose: TREATMENT	21 May 2009	23 May 2018
NCT00988741	Trial of ARQ 197 in Patients With Unresectable Hepatocellular Carcinoma (HCC) Who Have Failed One Prior Systemic Therapy	Completed	DRUG: ARQ 197|DRUG: Placebo	Evaluate time to progression among all patients treated with ARQ 197 compared to placebo. Patients will be evaluated every 6 weeks until unacceptable toxicity, disease progression or another discontinuation criterion is met	107	Interventional	Allocation: RANDOMIZED|Intervention Model: PARALLEL|Masking: QUADRUPLE (PARTICIPANT, CARE_PROVIDER, INVESTIGATOR, OUTCOMES_ASSESSOR)|Primary Purpose: TREATMENT	September 2009	March 2012
NCT06796803	Camrelizumab Combined with Rivoceranib and Hepatic Arterial Infusion Chemotherapy (HAIC) As Conversion Therapy for Potentially Resectable Hepatocellular Carcinoma(HCC)	Not_Yet_Recruiting	DRUG: camrelizumab combined with rivoceranib and HAIC|DRUG: camerlizumab + rivoceranib	R0 rate: R0 rate defined as the proportion of patients who accomplish the complete resection of tumor with pathologically confirmed negative margin, 24 months|OS, Overall survival (OS) after randomization, defined as the time from randomization to death from any cause, 24 months	398	Interventional	Allocation: RANDOMIZED|Intervention Model: PARALLEL|Masking: NONE|Primary Purpose: TREATMENT	20 February 2025	28 February 2030
NCT02989922	A Study to Evaluate SHR-1210 in Subjects With Advanced HCC	Completed	BIOLOGICAL: SHR-1210	Objective response rate: Tumour responses were evaluated by the independent review committee (IRC) according to RECIST 1.1. The primary endpoints were the proportion of patients with an IRC-assessed objective response (defined as the percentage of patients whose best overall response was confirmed complete or partial response); approximate 3 years|6-month overall survival rate, 6-month overall survival rate (defined as cumulative overall survival rate from the date of the first dose to 6 months), from the date of the first dose to 6 months	220	Interventional	Allocation: RANDOMIZED|Intervention Model: PARALLEL|Masking: NONE|Primary Purpose: TREATMENT	15 November 2016	3 March 2020
NCT05917431	Phase 2 Study of SBRT Plus Tislelizumab and Regorafenib in Unresectable or Oligometastatic HCC	Recruiting	COMBINATION_PRODUCT: SBRT plus tislelizumab and regorafenib	PFS, progression-free survival, from the date of treatment beginning until the date of first documented progression or date of death from any cause, whichever came first, assessed up to 48 months	39	Interventional	Allocation: NA|Intervention Model: SINGLE_GROUP|Masking: NONE|Primary Purpose: TREATMENT	June 2023	30 December 2026
NCT04732286	A Study of Atezolizumab in Combination With Bevacizumab in Spanish Patients With Unresectable or Unsuitable for Locoregional Treatments Hepatocellular Carcinoma Not Previously Treated With Systemic Therapy	Completed	DRUG: Atezolizumab|DRUG: Bevacizumab	Incidence of treatment discontinuations of atezolizumab and/or bevacizumab due to adverse events of grade ≥ 3, Initiation of study treatment up to approximately 3 years	100	Interventional	Allocation: NA|Intervention Model: SINGLE_GROUP|Masking: NONE|Primary Purpose: TREATMENT	4 May 2021	26 April 2024
NCT05733598	Study of RP2 in Combination With Second-line Therapy in Patients With Locally Advanced Unresectable or Metastatic HCC	Recruiting	BIOLOGICAL: RP2|BIOLOGICAL: Bevacizumab|BIOLOGICAL: Atezolizumab	Overall response rate per modified RECIST 1.1. The proportion of patients achieving a BOR of CR or PR per RECIST 1.1 as modified for use in this study among those that are evaluable for response., From Day 1 up to 3 years after first RP2 dose of last patient	30	Interventional	Allocation: NA|Intervention Model: SINGLE_GROUP|Masking: NONE|Primary Purpose: TREATMENT	1 August 2024	1 July 2028
NCT01755767	Study of Tivantinib in Subjects With Inoperable Hepatocellular Carcinoma (HCC) Who Have Been Treated With One Prior Therapy	Completed	DRUG: Tivantinib|DRUG: Placebo	Median overall survival (OS) following treatment with tivantinib 120 mg BID compared to placebo group in participants with MET diagnostic-high inoperable hepatocellular carcinoma (HCC) treated with one prior systemic therapy. Overall survival (OS) is defined as the time from randomization to the date of death. The rate of OS (percentage of participants still alive) was determined only in the tivantinib 120 mg BID cohort, within 36 months. Overall survival (OS) rate at different time points following treatment with tivantinib 120 mg BID compared to placebo group in participants with MET diagnostic-high inoperable hepatocellular carcinoma (HCC) treated with one prior systemic therapy. Overall survival (OS) is defined as the time from randomization to the date of death. The rate of OS (percentage of participants still alive) was determined only in the tivantinib 120 mg BID cohort, within 36 months	383	Interventional	Allocation: RANDOMIZED|Intervention Model: PARALLEL|Masking: QUADRUPLE (PARTICIPANT, CARE_PROVIDER, INVESTIGATOR, OUTCOMES_ASSESSOR)|Primary Purpose: TREATMENT	27 December 2012	31 July 2017
NCT00355238	A Phase II Open Label Study of BMS-582664 in Locally Advanced or Metastatic Hepatocellular Cancer	Completed	DRUG: brivanib (active)	Progression-free survival (PFS) rate at 6 months per Independent Response Review Committee (IRRC) in Cohort A. The percent of participants who have not progressed or died prior to 6 months from the date of their first dose. Participants who have neither progressed nor died but had their last tumor assessment prior to 6 months will not be categorized as progression free and will not be included. Tumor response was measured by the IRRC using mWHO criteria. Progression is defined as a 25% or more increase in the sum of all index lesion areas taking as reference the smallest sum recorded at or following baseline, from first dose up to approximately 6 months after first dose. The number of participants experiencing adverse events (AEs): An adverse event (AE) is defined as any new untoward medical occurrence in a participant or clinical investigation participant administered a pharmaceutical product and that does not necessarily have to have a causal relationship with this treatment, from first dose up to 30 days post last dose (up to approximately 34 months)	137	Interventional	Allocation: NA|Intervention Model: SINGLE_GROUP|Masking: NONE|Primary Purpose: TREATMENT	31 December 2006	30 April 2010
NCT04487067	A Study of Atezolizumab (Tecentriq) in Combination With Bevacizumab to Investigate Safety and Efficacy in Patients With Unresectable Hepatocellular Carcinoma Not Previously Treated With Systemic Therapy-Amethista	Completed	DRUG: Atezolizumab|DRUG: Bevacizumab	Number of participants with Grade 3–5 NCI CTCAE v.5 Bleeding/Haemorrhage, up to approximately 48 months	152	Interventional	Allocation: NA|Intervention Model: SINGLE_GROUP|Masking: NONE|Primary Purpose: TREATMENT	25 August 2020	13 August 2024
NCT06109272	A Study to Assess the Dose, Adverse Events, and Change in Disease Activity of Livmoniplimab as an Intravenous (IV) Solution in Combination With Budigalimab as an IV Solution in Adult Participants With Hepatocellular Carcinoma (HCC)	Active_Not_Recruiting	DRUG: Livmoniplimab|DRUG: Budigalimab|DRUG: Durvalumab|DRUG: Atezolizumab|DRUG: Bevacizumab|DRUG: Tremelimumab	Stage 1: Best overall response (BOR) per investigator: BOR is defined as a participant achieving confirmed complete response (CR) or confirmed partial response (PR) per Response Evaluation Criteria in Solid Tumors version 1.1 (RECIST 1.1) as determined by investigators at any time prior to subsequent anticancer therapy, through study completion, up to approximately 56 Months. Stage 2: Overall survival (OS): OS is defined as the time from randomization until death from any cause, through study completion, up to approximately 56 Months	660	Interventional	Allocation: RANDOMIZED|Intervention Model: SEQUENTIAL|Masking: NONE|Primary Purpose: TREATMENT	11 January 2024	September 2030
NCT06117891	An Observational Study to Learn More About How Well a Treatment Works When Given After Treatment With Atezolizumab and Bevacizumab or Another Similar Combination of Drugs in Adults With Liver Cancer That Cannot be Treated With Surgery	Recruiting	DRUG: Atezolizumab|DRUG: Bevacizumab|DRUG: Durvalumab|DRUG: Tremelimumab	Overall survival (OS): OS is defined as the time (days) from start of second-line systemic treatment to the date of death, due to any cause. Patients alive or lost to follow-up will be censored at their last date of follow-up; approximately 36 months	300	Observational	Observational Model: Time Perspective: p	27 November 2023	1 February 2027
NCT03764293	A Study to Evaluate SHR-1210 in Combination With Apatinib as First-Line Therapy in Patients With Advanced HCC	Completed	DRUG: SHR-1210|DRUG: Apatinib|DRUG: Sorafenib	Overall survival (OS): OS was defined as the time from randomization to death from any cause, up to approximately 3 years. Progression-free survival (PFS): Evaluated by the Blinded Independent Review Committee (BIRC) based on RECIST v1.1, PFS was defined as the time from randomization to the first occurrence of progressive disease (PD) by tumor image evaluation or death from any cause, whichever occurs first as determined by BIRC according to RECIST v1.1. PD: at least a 20% increase in the sum of diameters of target lesions and the sum of diameters must also demonstrate an absolute increase of \>/ = 5 millimeters (mm), or a measurable increase in a non-target lesion, or the appearance of new lesions, up to approximately 3 years	543	Interventional	Allocation: RANDOMIZED|Intervention Model: PARALLEL|Masking: NONE|Primary Purpose: TREATMENT	10 June 2019	14 June 2023
NCT06788353	Prospective Collection of Therapeutic Efficacy and Safety Data in Patients with Unresectable Hepatocellular Carcinoma	Recruiting	Not Provided	Progression-free survival, defined as the time from commencement of enrollment to progression of disease or death throughout the trial. Patients who withdraw or are lost to follow-ups will be treated as censored data, and the last date of known living without progression will be used as the last survival time. Patients whose diseases have not progressed at ending of this study will be treated as censored data, and the last date of known living without progression will be used as the last survival time, 24 months	500	Observational	Observational Model: Time Perspective: p	1 March 2023	1 March 2027
NCT03439891	Sorafenib and Nivolumab in Treating Participants With Unresectable, Locally Advanced or Metastatic Liver Cancer	Completed	BIOLOGICAL: Nivolumab|DRUG: Sorafenib	Maximum tolerated dose (MTD) (Part 1 Only): MTD is defined as the dose in which 1 or more dose limiting toxicities (DLTs) are reported by study participants in Part 1 within the first cycle of treatment. Participants in Part 1 must receive at least 2 doses of nivolumab and at least 75% of sorafenib doses within 28 days (1 cycle), or experience a qualifying DLT event to be evaluable, 28 days. Proportion of participants With grade 3 or higher treatment-related adverse events (Part 2 Only): All adverse events (AEs) will be summarized based on proportion of total participants in Part 2 to evaluate the safety of the treatment combination in participants with Child–Pugh B7-9 liver function as measured by proportion of participants with an AE of toxicity grade \> = 3 as graded by NCI Common Terminology Criteria for Adverse Events (CTCAE) version 4.03 and assessed as at least possibly related to sorafenib, nivolumab, or the combination of therapies, up to 2 years	16	Interventional	Allocation: NON_RANDOMIZED|Intervention Model: SEQUENTIAL|Masking: NONE|Primary Purpose: TREATMENT	16 April 2018	30 November 2023
NCT05717738	Combined TACE, TKI/Anti-VEGF and ICIs as Conversion Therapy for Advanced Hepatocellular Carcinoma	Recruiting	PROCEDURE: TACE|DRUG: Lenvatinib|DRUG: Anti-PD-1 monoclonal antibody|DRUG: Bevacizumab Biosimilar IBI305 plus sintilimab|DRUG: Bevacizumab plus Atezolizumab|DRUG: apatinib plus camrelizumab|DRUG: Sorafenib|DRUG: Donafenib|DRUG: Regorafenib	Number of patients amendable to curative surgical interventions: Number of patients amendable to curative surgical interventions defined as number of patients receiving curative surgical resection, transplantation, or ablation after successful down-sizing of tumor(s) by intervention., from the date of first treatment to the date of last treatment, an average of 3 years	300	Observational	Observational Model: Time Perspective: p	20 January 2022	31 December 2024
NCT05713994	Combined HAIC, TKI/anti-VEGF and ICIs As Conversion Therapy for Unresectable Hepatocellular Carcinoma	Recruiting	PROCEDURE: HAIC|DRUG: Bevacizumab plus Atezolizumab|DRUG: Bevacizumab Biosimilar IBI305 plus sintilimab|DRUG: Lenvatinib|DRUG: Sorafenib|DRUG: Donafenib|DRUG: Regorafenib|DRUG: apatinib plus camrelizumab|DRUG: Anti-PD-1 monoclonal antibody	Number of patients amendable to curative surgical interventions: Number of patients amendable to curative surgical interventions defined as number of patients receiving curative surgical resection, transplantation, or ablation after successful down-sizing of tumor(s) by intervention., from the date of first treatment to the date of last treatment, an average of 3 years	300	Observational	Observational Model: Time Perspective: p	19 May 2020	30 December 2025
NCT03782207	A Study Investigating the Outcomes and Safety of Atezolizumab Under Real-World Conditions in Patients Treated in Routine Clinical Practice	Active_Not_Recruiting	DRUG: Atezolizumab	Overall survival (OS): Time from index date until date of death from any cause. Index date is the date of administration of the first ever dose of atezolizumab for each patient, index date up to approximately 6 years. OS at 2 years: Percentage of participants alive 2 years after initiation of atezolizumab treatment, after index date up to 2 years	2756	Observational	Observational Model: Time Perspective: p	7 February 2019	31 July 2016
